# Strong long ties facilitate epidemic containment on mobility networks

**DOI:** 10.1093/pnasnexus/pgae515

**Published:** 2024-11-15

**Authors:** Jianhong Mou, Suoyi Tan, Juanjuan Zhang, Bin Sai, Mengning Wang, Bitao Dai, Bo-Wen Ming, Shan Liu, Zhen Jin, Guiquan Sun, Hongjie Yu, Xin Lu

**Affiliations:** College of Systems Engineering, National University of Defense Technology, Changsha 410073, China; College of Systems Engineering, National University of Defense Technology, Changsha 410073, China; Department of Epidemiology, School of Public Health, Key Laboratory of Public Health Safety, Ministry of Education, Fudan University, Shanghai 200032, China; Shanghai Institute of Infectious Disease and Biosecurity, Fudan University, Shanghai 200032, China; College of Systems Engineering, National University of Defense Technology, Changsha 410073, China; College of Systems Engineering, National University of Defense Technology, Changsha 410073, China; College of Systems Engineering, National University of Defense Technology, Changsha 410073, China; Department of Epidemiology, School of Public Health, Key Laboratory of Public Health Safety, Ministry of Education, Fudan University, Shanghai 200032, China; School of Management, Xi’an Jiaotong University, Xi’an 710049, China; Complex Systems Research Center, Shanxi University, Taiyuan 030006, Shanxi, China; Complex Systems Research Center, Shanxi University, Taiyuan 030006, Shanxi, China; Department of Mathematics, North University of China, Taiyuan 030051, Shanxi, China; Department of Epidemiology, School of Public Health, Key Laboratory of Public Health Safety, Ministry of Education, Fudan University, Shanghai 200032, China; Shanghai Institute of Infectious Disease and Biosecurity, Fudan University, Shanghai 200032, China; Department of Infectious Diseases, Huashan Hospital, Fudan University, Shanghai 200032, China; College of Systems Engineering, National University of Defense Technology, Changsha 410073, China

**Keywords:** strong long ties, epidemic containment, mobility networks, reaction–diffusion transmission model, grid-joint isolation strategy

## Abstract

The analysis of connection strengths and distances in the mobility network is pivotal for delineating critical pathways, particularly in the context of epidemic propagation. Local connections that link proximate districts typically exhibit strong weights. However, ties that bridge distant regions with high levels of interaction intensity, termed strong long (SL) ties, warrant increased scrutiny due to their potential to foster satellite epidemic clusters and extend the duration of pandemics. In this study, SL ties are identified as outliers on the joint distribution of distance and flow in the mobility network of Shanghai constructed from 1 km × 1 km high-resolution mobility data. We propose a grid-joint isolation strategy alongside a reaction–diffusion transmission model to assess the impact of SL ties on epidemic propagation. The findings indicate that regions connected by SL ties exhibit a small spatial autocorrelation and display a temporal similarity pattern in disease transmission. Grid-joint isolation based on SL ties reduces cumulative infections by an average of 17.1% compared with other types of ties. This work highlights the necessity of identifying and targeting potentially infected remote areas for spatially focused interventions, thereby enriching our comprehension and management of epidemic dynamics.

**Significance Statement**

This study illuminates the differentiation between the strength and length of connections on mobility networks. Our research reveals that connections bridging distant regions with high interaction intensity, termed strong long (SL) ties, exhibit low spatial autocorrelation and display a temporal similarity pattern in disease transmission, potentially fostering satellite epidemic clusters and extending the duration of pandemics. The superiority of grid-joint isolation strategy based on SL ties suggests that the adoption of advanced isolation strategies targeting remote grids, which maintain high-flow connections to infected grids, is imperative for the formulation of effective policies. Our work provides different perspectives for assessing the role of SL ties in network dynamics, deepening our comprehension of various realms, including economics, social, and biological systems.

## Introduction

Although ties serve as crucial channels in epidemic spreading, their impacts vary depending on the type of ties involved, including positive and negative edges on signed networks (1), and inter-community links on community-related networks (2). Moreover, several studies highlight the decisive role of intensity in shaping epidemic propagation in mobility networks (3, 4). Research across various disciplines has extensively examined the impacts of long ties (LG), focusing on their spatial extent (5–7) and tie range (8, 9). These studies collectively highlight the fundamental role of LG in bridging crucial structures across various networks. LG serve as critical channels for the rapid dissemination of novel information and the spread of contagious behaviors, underscoring their significance in understanding and managing the dynamics of social (10), biological (11), and epidemiological phenomena (12–15). In the context of human mobility, infectious diseases frequently transcend localized geographical regions, spreading rapidly across countries and continents. This proliferation is largely facilitated by the movement of infected individuals over large spatial scales (4, 16–18). Such long-distance dispersal is generally anticipated to accelerate the viral spread within an extensive population (19). Once an infected individual travels from the primary outbreak spot to an unaffected remote one, it triggers the emergence of a new infectious subpopulation, known as a satellite cluster. These nascent clusters can subsequently expand, potentially serving as the source for further long-distance spread of the disease. The limited overlap between the initial outbreak and satellite clusters often results in more extensive infections across a wider spatial scope.

LG are often structurally categorized as weak ties (20–22), providing unique informational benefits not readily available through closer contacts (23–25). Strong ties (ST), regarding the topological proximity of associated nodes, are theoretically proven to facilitate the epidemic prevalence (3). However, their significance in mobility contexts, particularly in urban environments, necessitates a reevaluation of their perceived weakness and a distinction between the length and strength of ties (26). High mobility across distant locations facilitated by point-to-point transportation underscores the significance of these ties over extended spans (27, 28). It has been highly recommended that these travels be retained as much as possible to extract spatial network structure from large-scale origin–destination flow data (29). Notably, in some instances, high traffic volumes on long-distance routes, such as those connecting airports and rail stations, surpass those of nearby travel. The efficacy of public transportation shutdowns, specifically long-distance buses, in mitigating the spread of a citywide epidemic (30), underscores the pivotal role of long-distance ties characterized by significant mobility, referred to as strong long (SL) ties. Severing these ties aids in containing diseases within localized areas, thereby accelerating epidemic extinction, especially when detailed trajectories of infected individuals are unknown. Mobile device data, especially derived from call detail records (31–33), facilitates the identification of SL ties and aids in understanding epidemic propagation on a citywide scale. However, a majority of studies (34, 35) employ mobility data with low spatial resolution, ranging from a few to dozens of kilometers, limiting the in-depth investigation needed to distinguish the length and strength of mobility ties.

Numerous researchers have confirmed the effectiveness of nonpharmaceutical interventions (NPIs) in controlling the spread of physical-contact diseases, including social network-based distancing (36), contact tracing (37), citywide lockdown (38), and et al. The impact of community structure on epidemics offers valuable insights for preventing disease spread between communities by changing the structure of the contact network through NPIs (39, 40). Inter-community ties, one of the types of LG, play a crucial role in epidemic control for the profound impact of community structure on network dynamics, yielding the outperformance of immunizing interventions targeted at individuals bridging communities compared with those targeting highly connected individuals (40). However, these approaches may underestimate the risk posed by unconfirmed infected individuals traveling to distant districts, which could potentially facilitate further transmission. The strength of LG is also vital, because it serves as a critical indicator for identifying remote areas where unconfirmed infected individuals might be located, thereby aiding in the preemptive containment of potential infection hotspots. Despite its significance, limited research has been conducted on the strength of LG, particularly in differentiating between LG and SL ties, let alone the quantification and identification of SL ties. Additionally, it remains uncertain whether the vitality of grids connected to the initial outbreak via SL ties exceeds that of grids connected by other types of ties. This raises a critical decision point for policymakers: whether limiting restrictions to geographical neighboring areas suffices, or if it is necessary to expand containment efforts to remote regions connected by SL ties. This uncertainty underscores the need for a nuanced analysis of human mobility patterns and their influence on disease spread.

This work emphasizes connections that possess both spatial and social characteristics, taking into account the spatial heterogeneity of the intensity distribution, rather than focusing solely on single attributes like the edge position or signed attributes. We also intend to thoroughly evaluate the significance of SL ties in identifying potentially hazardous areas. Specifically, grids connected by SL ties to the initial outbreak site are more likely to form satellite clusters, given the positive correlation between transmission probability and population flow. Initially, we quantify and characterize SL ties for each 1 km × 1 km grid in Shanghai by aggregating distance and population flow from high-resolution cellular signaling data (CSD). We propose a grid-joint isolation strategy that supports preemptive quarantine for unconfirmed grids receiving individuals from confirmed ones, in which a reaction–diffusion transmission (RDT) model is established to simulate the spread of the Omicron variant of SARS-CoV-2 cross grids in Shanghai. This strategy facilitates quantifying the effectiveness of SL ties in identifying potential high-risk grids compared with ties defined by other criteria: those with the highest flow (ST), the greatest distance (LG), and the shortest distance (short ties, SH).

## Results

### Statistical and spatial features of ties

**Fig. 1.**
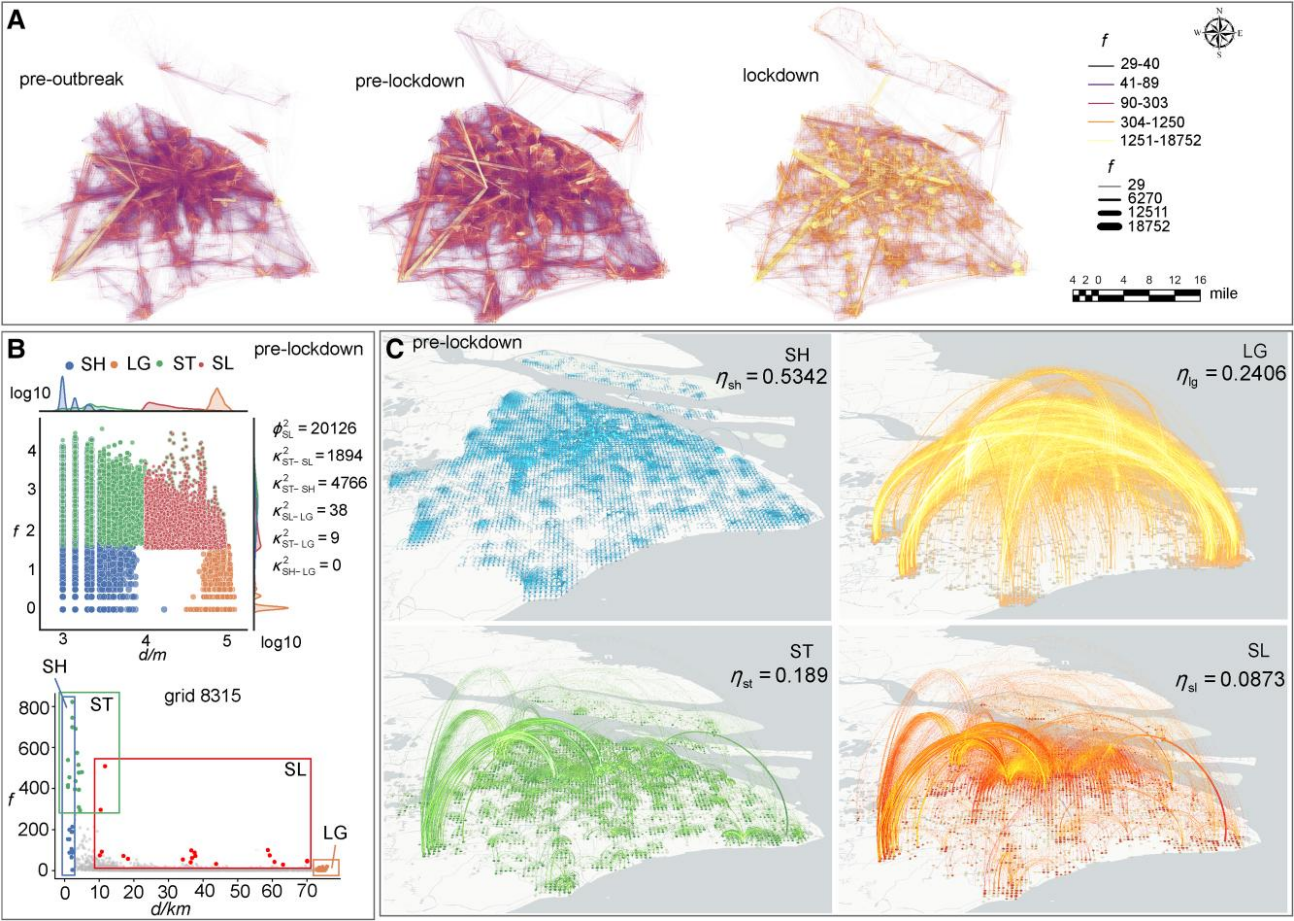
Statistical and spatial characteristics of various types of ties. Although different types of ties share several connections, they show various statistical and spatial characteristics. A) The overviews of mobility patterns during three phases are illustrated. B) The joint distribution of flow (*f*) and distance (*d*) for various types of ties during the prelockdown phase with an example of grid 8,315 is shown. The number of all types of ties for each grid keeps in line with that of SLs. $\phi_{\mathrm{SL}}^{2}$ is the number of SLs during the second phase and $\kappa_{\mathrm{ST}\text{-}\mathrm{SL}}^{2}$ represents the number of overlapping ties between STs and SLs. C) The spatial distribution of SHs, LGs, STs, and SLs along with Moran's indices *η*. Linewidth represents the strength of the connection is exhibited.

The overview of mobility patterns during three phases (see Fig. 1A and Table S1), segmented according to the intervention stringency (see Materials and methods for details), illustrates the prevalence of long-distance connections with heavy flow, i.e. SLs. This type of tie is quantitatively identified as outliers using DBSCAN with a baseline of distance larger than 10 km (d>10,000) and flow greater than 28 (f>28) (see Materials and methods for details). The flow of connections refers to the number of individuals traveling through during the specific phase.

To differentiate the characteristics of SLs from STs, LGs, and SHs, we define a fixed number of ties, *k*, as that of SLs for each grid. Specifically, we respectively extract ties characterized by the top-*k* maximum flow, maximum distance, and minimum distance. As shown in Fig. 1B, during the prelockdown phase (2022 March 1–31), 23.68 and 9.41% of STs are also SHs and SLs, respectively, whereas only 0.19% of SLs are identified as LGs. Statistically, STs predominantly concentrate on grid pairs with average distance $\bar{d}=5,656.93$ and average flow $\bar{\;f}=810.65$, with outliers at long distances. SLs concentrate on outliers with a relatively narrow flow distribution. LGs, covering distances from 50 to 100 km, typically demonstrate flows under 200. Conversely, SHs, excluding self-connections, focus on local connections within a 10 km radius, with an average flow $\bar{\;f}=404.92$ (see Fig. S3). SHs and STs significantly overlap because short-distance ties tend to generate heavy flow. STs and SLs show minor overlap since several long-distance ties accommodate larger populations than their short-distance counterparts.

Spatially, these four types of ties can be distinguished through spatial autocorrelation, quantified by the global Moran's index *η*, which measures the similarity of incoming connections among neighboring grids (see Supplementary Note S1). The incoming connections of grids refer to the possibility being passively isolated imposed by the infected neighbors, and the autocorrelation is highly related to the overlap of neighbors for all nodes. SLs show the lowest spatial autocorrelation, with an average Moran's index ${\bar{\eta }}_{\mathrm{sl}}=0.0974$ across three phases. Grids linked by SHs display a spatially clustered distribution, resulting in the highest average autocorrelation ${\bar{\eta }}_{\mathrm{sh}}=0.5615$, followed by STs and LGs with ${\bar{\eta }}_{\mathrm{st}}=0.2536$ and ${\bar{\eta }}_{\mathrm{lg}}=0.1935$, respectively (see Table S2). All spatial autocorrelations are statistically significant with *P*-value <0.001. Grids connected by SLs are randomly distributed without significant clustering, whereas SHs connect spatially proximate grids, showing similar incoming connections. Peripheral grids connected by LGs show significant clustering, but grids outside these clusters are scattered, thereby reducing spatial autocorrelation (see Figs. 1C, S4, and S5).

### Epidemic temporal similarity among grids

**Fig. 2.**
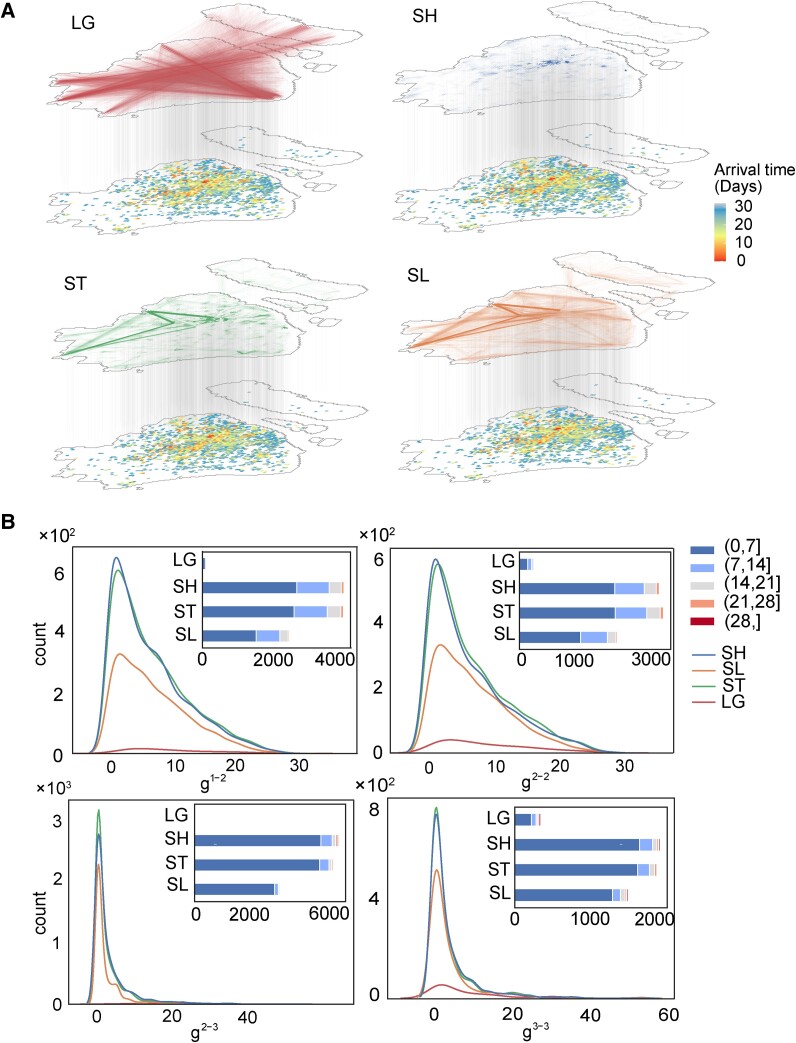
The effect of different types of ties on capturing temporarily highly related grids. SL effectively identifies grids with substantial flow across extensive spatial distances and preserves the temporal concordance of grid pairs. A) The spatial distribution of arrival time during the prelockdown phase for grid pairs connected by various types of ties in the same phase is exemplified. The linewidth of connections on the top mobility network represents the population flow, and the color of grids on the bottom map denotes the arrival time. B) The statistical distribution of the gap of arrival time between grid pairs, denoted as *g*, with insets displaying the cumulative histograms for each type of ties in the unit of 7 days is indicated. Noticeably, the nonsignificant mobility variation between the preoutbreak and prelockdown phases brings in a similar distribution between $g^{1-2}$ and $g^{2-2}$, and the citywide epidemic propagation during the prelockdown phase causes the concentration of $g^{2-3}$ and $g^{3-3}$.

Most grids show a temporal correlation during epidemic propagation, indicating that grid pairs may become infected within a short time. Identifying these grid pairs and severing their connection can be instrumental in reducing the highly dynamic correlation, thereby slowing disease transmission. The length of SLs is crucial in facilitating the spread of the epidemic between distant grid pairs. The strength of these connections reflects the temporal concordance of arrival times between such grid pairs, as the probability of transmission between grids is contingent upon the population flow. This study investigates the variation in arrival times between grid pairs to evaluate the temporal similarities in epidemic spread. To figure out the relationship between human mobility and temporal similarity, i.e. whether the temporal similarity is caused by previous or current human mobility, we examine the gap of arrival times during the prelockdown and lockdown (2022 April 1–2022 May 30) phases for grid pairs connected by ties in the previous stages: the preoutbreak (2022 February 15–28) and prelockdown phases, denoted as $g^{1-2}$ and $g^{2-3}$, respectively. The discrepancies within the same stages are also discussed, denoted as $g^{2-2}$ and $g^{3-3}$ (see Fig. 2A). Specifically, $g^{x-y}$ is the set of $g_{ij}^{x-y}$ overall connections under the considered phases, i.e. $g^{x-y}=\{g_{ij}^{x-y}\}$, where $g_{ij}^{x-y}=|t_{i}^{y}-t_{j}^{y}|,(i,j)\in E^{x}$ and $t_{i}^{y}$ represents the arrival time of grid *i* which is infected within the phase *y*, and denotes the different types of ties during phase *x*.

The findings suggest that SLs effectively identify grids with substantial flow across extensive spatial distances and preserve the temporal concordance of grid pairs, akin to STs and SHs (see Fig. 2B). Specifically, SLs capture, on average, 71.77 and 61.39% of grid pairs with an arrival time gap of <7 days, compared with STs within the current and previous phases, respectively (see Table S3). The increasing number of grid pairs with $g^{1-2}\leq 7$ and $g^{2-3}\leq 7$ compared with those with $g^{2-2}\leq 7$ and $g^{3-3}\leq 7$ supports the guiding effect of previous mobility on epidemic temporal similarity. Temporally similar and spatially remote grids from the initial outbreak site significantly accelerate epidemic propagation, as they get infected quickly and trigger further epidemic expansion with little overlap with the initial one. This underscores the importance of implementing preemptive quarantine measures on grids connected to the initial outbreak grid by SLs to mitigate epidemic propagation.

### Epidemic propagation containment based on grid-joint isolation strategy

**Fig. 3.**
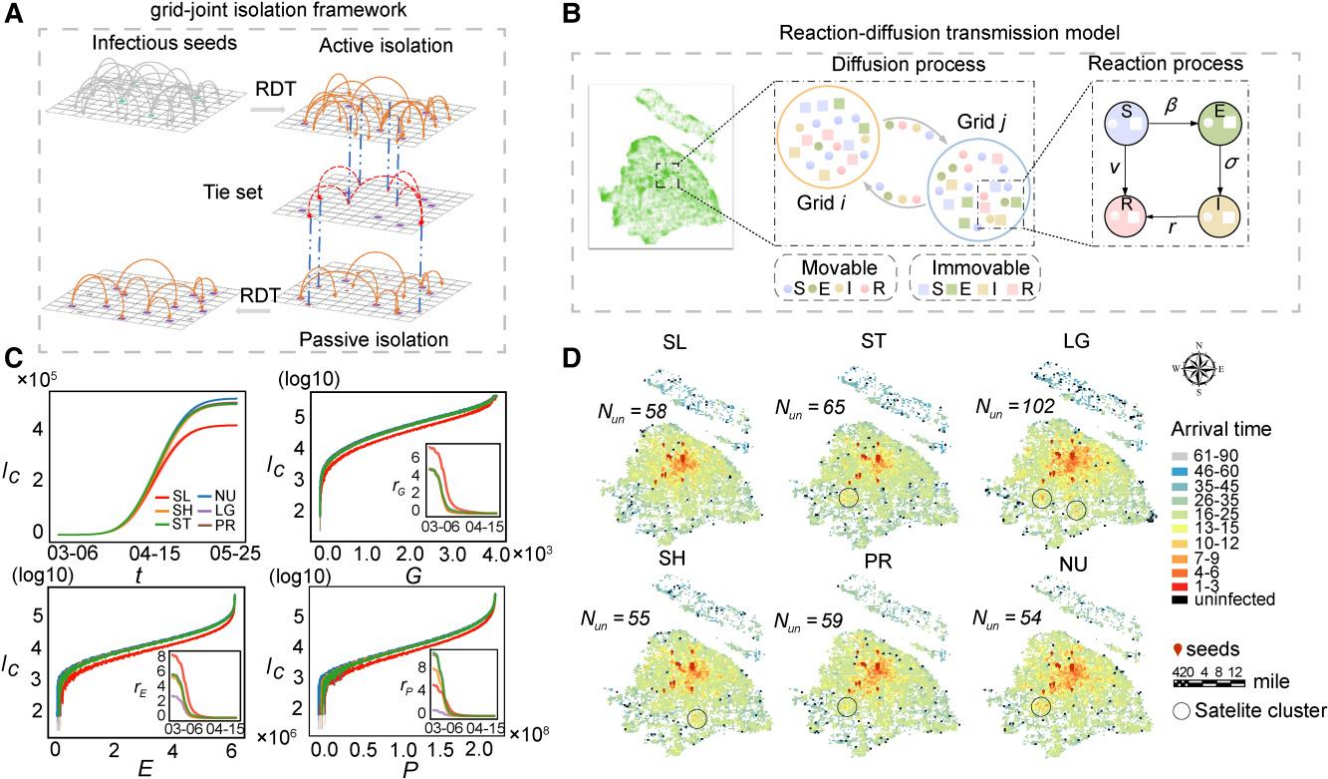
The effect of SL ties on reducing contagions. Grid-joint isolation based on SLs slows down epidemic propagation for its positive effect on isolating remote satellite clusters. A) The general framework of grid-joint isolation is displayed. The initial infectious grids will be actively isolated if infections surpass the threshold *α*, and the unconfirmed grids (stars) pointed by other actively isolated girds with tie set (dashed line) will be passively isolated with the elimination of related connections. Squares represent unconfirmed grids, i.e. girds with patients < *α*. The epidemic propagation across spatial networks is ruled by the RDT. B) The schematic illustration of the RDT model where individuals change their states according to the SEIR mechanism within each grid and diffuse between grids subject to the population flow is shown. Circles and squares respectively denote the movable and immovable individuals with S, E, I, and R states, differentiating with different colors. C) The performance of SLs in reducing the cumulative infections ($I_{c}$) along with the corresponding number of isolated grids (*G*), restricted edges (*E*), and quarantined population (*P*) is illustrated. Results are averaged over 50 independent realizations. $r_{G}$, $r_{E}$, and $r_{P}$, respectively denotes the relative improvement compared with NU in terms of isolated grids, restricted edges, and quarantined population. D) The epidemic arrival time of each grid after grid-joint isolation through various types of ties, where circles indicate satellite clusters is exhibited. The number of uninfected grids for each strategy ($N_{\mathrm{un}}$) is given.

The ability of SLs to identify remote grid pairs with high temporal similarity highlights the potential effectiveness of preemptively isolating neighboring grids connected by these ties, as described by the grid-joint isolated strategy in this work (see Fig. 3A and Materials and methods). This strategy supports simultaneous preemptive isolation of neighbors (passive isolation) of grids that are confirmed infectious and directly isolated (active isolation). The infectious grids are confirmed once the number of patients exceeds a certain threshold *α*. Specifically, the threshold *α* represents the criterion of isolating grids, thus describing the strictness of epidemic control for each grid. The RDT model (Fig. 3B and see Materials and methods) simulates epidemic propagation across grids during grid-joint isolation with the goodness of fit equaling 0.93, as validated in Fig. S6. To minimize the impact of infection dynamics on grids not linked to SLs, we developed a new mobility network comprising 4,358 SL-related grids and their corresponding 6,172,854 population flows. Additionally, to evaluate the relative effectiveness of different tie types in reducing infections, we standardized the number of controllable neighbors for each grid to match the SL scenario. In this section, we also consider grid-joint isolation according to the infection pressure (PR) of grids which describes the probability of the grid being infected (see Supplementary Note S2 for details). The strategy of passively isolating grids linked by SLs results in a relative reduction in cumulative infections by average 17.1%, i.e. $\left\langle R_{d}\right\rangle =0.171$, compared with other types of ties, and an even greater reduction, $R_{d}=0.196$, compared with a null model (NU) that only isolates confirmed grids (see Table S4 and Fig. 3C). Notably, although the SL-based isolation strategy brings in more isolated grids and restricted routines compared with other types of ties, it yields less quarantined individuals than SH, ST, and PR.

Simulations involving grid-joint isolation through all types of ties, except SLs, reveal the emergence of several significant remote satellite clusters that are infected early (see Fig. 3D). These clusters act as new sources of outbreak, accelerating the spread of the epidemic. In addition, SL-based grid-joint isolation yields a similar number of uninfected grids compared with other types of ties, except for LGs. Since grids related to LG are usually the city peripheries that are typically infected lately, they are often passively isolated at the time earlier than the possible infected times, thus increasing the isolation of uninfected grids. It is noteworthy that simulation under STs yields fewer grids within a short arrival time than SLs. Specifically, the ST-focused isolation strategy leads to 853 grids with an arrival time of <14 days, which is fewer than that achieved through SL-oriented isolation, and significantly less than the 1,324 grids observed in the NU scenario (see Fig. S7). To explain the discrepancy between the cumulative infections and the arrival time associated with SLs, we examine the dynamics of daily confirmed infections under different control measures (see Fig. S8). In the context of STs, several grids with arrival time smaller than 14 days are identified as satellite seeds and initiate new outbreaks, raising the number of grids with an arrival time of 25–34 days. The reaction process of the RDT model within these grids induces increasing infections after several days. Consequently, satellite clusters become primary drivers of secondary propagation, leading to heavy daily confirmed infections. From the perspective of community structure in mobility networks, SLs capture more inter-community links than STs and SHs, thereby isolating more grids with diverse topological features under the joint-grid isolation strategy. Although LGs connect grids across different communities, most passively isolated grids are located at city peripheries without any patients, thus diminishing the effectiveness of passive isolation (see Supplementary Note S3 and Fig. S1).

### Effective intervention of SL ties

**Fig. 4.**
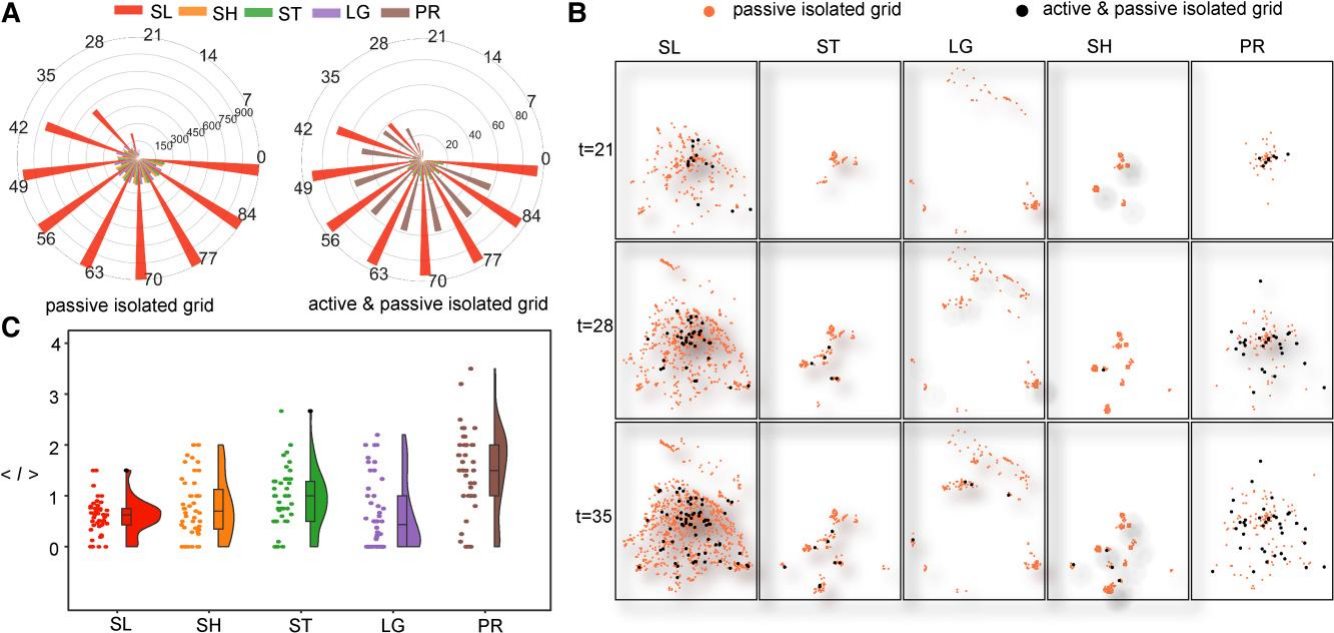
The effectiveness of passive isolation. SLs yield more passive isolated girds and isolate grids earlier than other types of ties. A) The distribution of the number of passive isolated grids and active and passive isolated grids at the step of 7 days is shown. The ticks on the circle are the propagation time. B) The spatial distribution of passive isolations at several times is illustrated. C) The average infected individuals of grids <l> when they are passively isolated under the grid-joint isolation strategy based on various types of ties are displayed.

The deep analysis of what happened during the grid-joint isolation process facilitates a comprehensive understanding of the performance of various types of ties. Since actively isolated grids may simultaneously opt for passive isolation of their shared neighboring grids, the grid-joint isolation strategy favors grid pairs with few common neighbors. The small spatial autocorrelation of SL explains the significant superiority of capturing more passive isolation. Considering the passive isolation of grids with patients larger than *α* since they are pointed by other actively isolated grids, i.e. active and passive isolated grids, SL and PR facilitate the identification of highly hazardous grids (see Fig. 4A).

SLs tend to passively isolate sparsely distributed grids due to their low spatial autocorrelation, whereas other types of ties might isolate the identical grid through different links due to their high Moran's index. The overlap reduces the number of passively isolated grids, thereby diminishing the efficiency of quarantine measures under the assumption that various isolated grids can concurrently and independently select their common neighboring grids for passive isolation within the grid-joint isolation strategy. The nonoverlapping passively isolated grids through SLs prevent disease transmission to spatially scattered districts, limiting disease spread within geographical proximity and reducing the number of active isolated grids. In contrast, other types of ties typically confine passive isolation to several clustered localities, thus exerting minimal impact on mitigating overall propagation and, consequently, necessitating increased active isolation (see Fig. 4B).

To examine the efficiency of passive isolation, we check the average infected individuals when grids are passively isolated. Figure 4C indicates that SL yields passive isolation when grids are usually slightly infected, i.e. with few patients. It supports the early intervention of the SL-based strategy compared with other types of ties. Notably, the passively isolated grids of LG are usually city peripheries that are uninfected when they are isolated, thus lowering the average number of infections.

### Sensitivity analysis

#### The effect of criteria for SLs and grid isolation

**Fig. 5.**
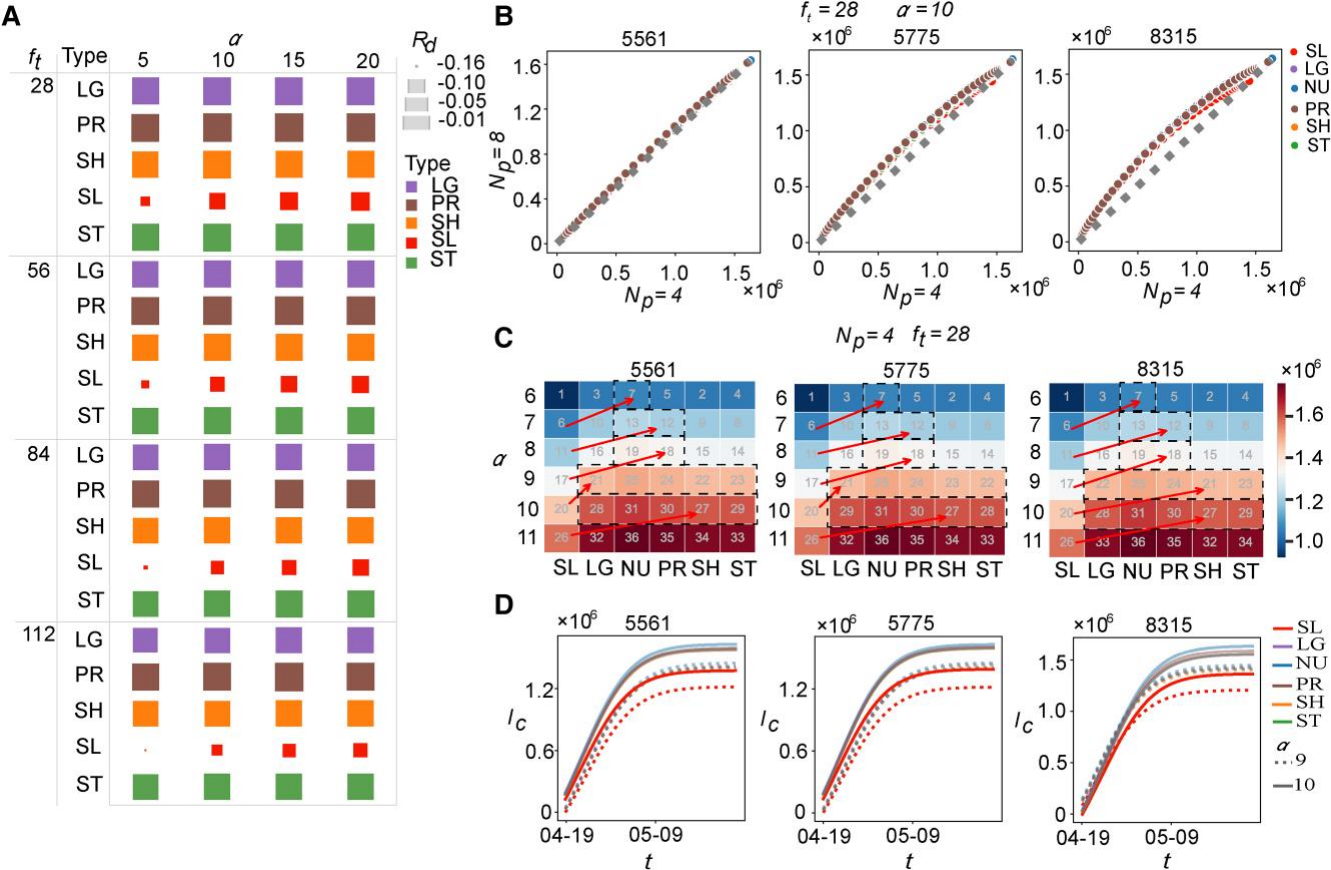
Sensitivity analysis of SL ties on slowing down pandemics. Grid-joint isolation strategies focusing on SLs demonstrate superior efficacy in mitigating epidemic propagation, irrespective of variations in $f_{t}$ and *α*. In addition, various initial infection grids and the number of patients make little difference in the outperformance of SLs. More importantly, the SL-based grid-joint isolation strategy yields fewer infections under loose restriction compared with other types of ties under rigorous restriction. A) The relative improvement ($R_{d}$) of cumulative infections of various types of ties over NU with different flow thresholds ($f_{t}$) and isolation threshold (*α*) is illustrated. The size of square is upon to $R_{d}$. B) The results of epidemic propagation originating from grid 5,561, 5,775, and 8,315 with different initial patients $N_{p}$ when α=10 are shown. C) The effect of the isolation threshold *α* on the performance of SLs originating from distinct grids with four patients is illustrated. The color maps the cumulative infections, and the number inside the rectangle indicates the ascending order. The arrows represent the ascending order from SLs to others, and dashed lines show the cases where SLs with loose restriction outperform others with rigorous restriction. D) The cases where SLs at α=10 outperform all other types of ties at α=9 are detailed.

This section evaluates the impact of SLs and grid isolation criteria on the efficacy of pandemic mitigation strategies across varying flow threshold $f_{t}$ and isolation threshold *α*. Results indicate that grid-joint isolation strategies targeting SLs consistently outperform other methods (see Fig. 5A). Specifically, a smaller *α* significantly enhances the effectiveness of SLs in slowing pandemic spread. The observed decline in performance with increasing *α* underscores the critical role of SL-based grid-joint isolation in curbing epidemic propagation in the early stages. The distinction between SLs and other types of ties becomes less pronounced as the epidemic proliferates. In addition, although an increasing $f_{t}$ reduces the number of SLs, it demonstrates the heightened efficiency of SLs. Constructing a smaller mobility network from fewer SLs characterized by high flow essentially distills the original network to its core structure, thereby accentuating the advantage of strategy focusing on SLs.

#### The effect of outbreak grid and initial infections

We further investigate how performance changes with different outbreaking grids and initial infections $N_{p}$. Specifically, we measure cumulative infections originating from a single grid with different characteristics, including that with few nonsignificant SLs, several SLs showing larger flow than SHs, and significant SLs illustrating smaller flow than SHs, i.e. grid 5,561, 5,775, and 8,315, respectively (see Fig. S9 for details). Epidemic propagation from each seed with various patients indicates that SLs are always beneficial for identifying potential unconfirmed grids and reducing infections, regardless of spatial features and initial infections (see Fig. 5B). Additionally, the number of initial patients has little influence on the spreading dynamics from grid 5,561, while more patients accelerate the propagation from grid 5,775 and 8,315. Interestingly, the propagation dynamics from the single seed with four patients show that the grid-joint isolation strategy based on SLs with large *α* usually results in fewer infections than other types of ties with small *α* for all grid types (see Fig. 5C), especially the comparison of SLs with α=10 and others with α=9 (see Fig. 5D). This finding highlights that the SL-oriented control measure yields fewer infections with loose restrictions compared with other types of ties with rigorous restrictions.

## Discussion

In this study, we refine our understanding of mobility networks by categorizing connections based on their distance and flow, specifically identifying SL ties as those linking distant districts with substantial traffic. These ties, characterized by their significant flow over long distances, stand out as anomalies in the distance-flow dimension, and are precisely detected using the DBSCAN algorithm. Unlike other types of ties, SL ties exhibit lower spatial autocorrelation and capture the significant temporarily similarity across distant regions, potentially acting as the satellite seeds and inducing infection clusters. Our exploration into the impact of these ties on epidemic spread offers invaluable insights into identifying critical areas for targeted interventions and implementing effective control strategies. We proposed a grid-joint isolation strategy to facilitate the understanding of epidemic dynamics affected by the spatial characteristics of various types of ties. Our findings compellingly suggest that the adoption of advanced isolation strategies targeting remote grids, which maintain high-flow connections to infected grids, is imperative for the formulation of effective policies. This assertion holds irrespective of the identification of SL ties, the threshold for grid isolation, the spatial feature of grids, and the number of initial patients. Furthermore, under the circumstance of a single infection source, SLs illustrate fewer infections with loose restriction than other types of ties with rigorous restriction.

Population mobility at different scales is influenced by a variety of complex factors. Within a city, grids are heterogeneous in terms of citizen service due to the scattered distribution of points of interest (POIs). To address this inequality, numerous transportation are available at lower costs, facilitating remote travel between schools, hospitals, subway stations, and airports. On a national or even international scale, the adequate infrastructure of each city, ensuring homogeneity in basic living standards, reduces the necessity for most people to travel between cities hundreds of miles apart, except for business or leisure purposes. Although Simpson's Paradox between the significance of SL between city-scale and national-level mobility networks (see Supplementary Note S4 and Fig. S2) underscores the essentiality of our work for targeted interventions, the innovative definitions and identification of outlier connections in macroscopic mobility networks and their impact on spreading dynamics are worthy of investigation. On the potential factors yielding different spatial distributions of SL ties, the heterogeneous distribution of POIs, the spatial distribution of citizens, and the layout of cities may be the next in-depth investigation for understanding the intrinsic motivations of SL ties. Additionally, quarantine resources are usually limited to offer the long-term isolation of grids and restriction of individual mobility, which necessitates the consideration of grid de-isolation. Although the grid-joint isolation strategy has compensated for the negligibility of severing SL ties on a highly connected mobility network, the effect of cutting or altering links on epidemic propagation in mobility networks with various topology and mobility characteristics may further support the effective containment by cutting the critical connections.

## Materials and methods

### Data sources

#### High-resolution CSD

The CSD data provided by China Unicom, one of China's major national mobile carriers, captures the activities of roughly a third of Shanghai's active mobile phone users. This data collection spans both active and passive events: active signaling occurs during phone calls, texts, device power cycles, or tower transitions, while passive signaling logs user locations at regular intervals (about every 30 min) without requiring active phone use. Detailed in the CSD are specifics like event timing, user demographics, and the start and end points of their movements across grid locations. Our methodology aggregates this information, preserving anonymity and privacy while providing a basis for our analysis.

#### The mobility network of Shanghai

Utilizing CSD data, we construct the mobility network based on population movements across 1 km × 1 km grids. A movement is logged when a user switches to a new cell tower, until the user becomes stationary again, i.e. without switching to another tower for roughly 30 min. In this network, 7,355 grids act as nodes, and the 7,819,894 population flows between these grids are represented as directed and weighted edges. Furthermore, self-connections on nodes signify intra-grid movements, reflecting mobility within the identical grid.

#### Daily new confirmed infections of SARS-CoV-2 Omicron in Shanghai

This study collects data on a daily basis, aggregating infection counts along with detailed case information for both asymptomatic and symptomatic instances of SARS-CoV-2. The data is sourced from multiple publicly accessible official sources, including municipal health commission websites and local government media outlets, with details in referenced materials (41).

#### Timeline of the outbreak and public health response

Acknowledging the role of NPIs in curtailing population movement (42, 43), we segment the study period into three distinct phases, as defined by the Stringency Index from the Oxford COVID-19 Government Response Tracker (44, 45). The initial phase, preoutbreak, covers 2022 February 15 to 2022 February 28, characterized by minimal impact on people's daily activity. Subsequently, the prelockdown phase begins on March 1, characterized by the activation of various NPIs aimed at controlling the epidemic spread. Finally, the lockdown phase, from April 1 to May 30, witnesses the comprehensive travel restrictions across the city, significantly limiting mobility.

### Identifying SL ties

SLs are defined as connections characterized by high population flow over long distances. Given that trips exceeding 10 km constituted 33.3% of all travel in Shanghai during the preoutbreak period, with individuals making an average of 1.36 trips daily (46), we establish a baseline for SLs as those exceeding 10 km and having a flow greater than 28 assuming that individuals make at least one trip daily. For quantitative identification, we categorize SLs as connections with a flow exceeding that of spatially proximate ties from the same origin. These are identified as outliers of the joint distribution of distance and flow. Considering the superiority of DBSCAN (47) in handling datasets with undefined clusters and varying shapes, particularly its effectiveness in anomaly detection, we identify all significant SL ties for each grid by setting ε=0.02 and s=5 (see Supplementary Note S5 for details). Although SLs are outliers in geographical proximity, they frequently exhibit higher flow than SHs, as shown in Fig. S10. In practice, it is typical for many individuals to travel directly to distant locations from specific points, whereas only a minority wander in the nearby vicinity.

### RDT model

Although a variety of network models have been developed to explain and predict the complex contagion dynamics, including multiplex networks (48, 49), hypergraphs (50, 51), and higher-order networks (52), we model the epidemic propagation related to human mobility as a reaction–diffusion process. It is commonly (53–55) employed to model a variety of phenomena including chemical reactions, population dynamics, epidemic spread, and other spatially distributed systems where particles diffuse in space and are subject to various reaction processes. In citywide meta-population epidemic scenarios, the reaction process models the likelihood of individuals coming into contact and altering their health status based on the infection dynamics within the same location or compartment. The diffusion process relies on the movement patterns within grid-based mobility networks, with only mobile individuals contributing to the spatial propagation of the epidemic.

Considering the incubation period of SARS-CoV-2, the dynamics of COVID-19 infection within compartments are predominantly captured by the SEIR model (37, 56), which categorizes individuals into states: susceptible (S), exposed (E), infectious (I), and removed (R). Patient zero transmits the infection to others under the SEIR mechanism. Following the reaction phase, individuals move from grid *i* to grid *j* based on the movement probability $P_{ij}=F_{ij}/\sum_{k\in \Phi (i)}F_{ik}$, where $F_{ij}$ represents the population flows from *i* to *j* and Φ(i) denotes the neighboring grid of *i*. The immobile individuals within a grid are represented by $F_{ii}$. Before confirmation and isolation, the newly infected individuals contribute to subsequent diffusion, resulting in sustained propagation.

#### Reaction process

Upon encountering an infectious agent, a susceptible individual transitions to the exposed category with a specified transmission probability *β*, subsequently advancing to the infectious state at a given incubation rate *σ*, and ultimately being removed from the population at a rate denoted by *r*. Furthermore, the probability of immunity, denoted by *v*, accounts for individuals within grids who have survived infection. Each grid is represented as a subpopulation where individuals are assumed to be uniformly mixed and the infection process approximates the following deterministic and continuous-time model:


(1) $$\begin{array}{rl} \frac{dS_{i}}{\mathrm{dt}} & =S_{i}-\frac{\beta \times S_{i}\times I_{i}}{N_{i}}-v\times S_{i}\\ \frac{dE_{i}}{\mathrm{dt}} & =(1-\sigma )\times E_{i}+\frac{\beta \times S_{i}\times I_{i}}{N_{i}}\\ \frac{dI_{i}}{\mathrm{dt}} & =(1-r)\times I_{i}+\sigma \times E_{i}\\ \frac{dR_{i}}{\mathrm{dt}} & =r\times I_{i}+R_{i}+v\times S_{i} \end{array}$$

#### Diffusion process

Following the intra-grid reactions, individuals engage in inter-grid commuting, adhering to predefined origin–destination routes. In this work, we assume that individuals confirmed as infectious will be quickly quarantined, thus the movement of infectious individuals primarily pertains to those who remain undetected. To distinguish between the populations that are movable and immovable, we introduce an exit probability *D*, reflecting the propensity of individuals to depart from grids. Consequently, after the diffusion process, the population within grid *i* consists of both residual individuals and those arriving from other grids, which follows:


(2) $$\begin{array}{rl} S_{i}(t+1) & =D\sum_{\;j=1}^{N}P_{\;ji}S_{j}(t)+(1-D)S_{i}(t)\\ E_{i}(t+1) & =D\sum_{\;j=1}^{N}P_{\;ji}E_{j}(t)+(1-D)E_{i}(t)\\ I_{i}(t+1) & =D\sum_{\;j=1}^{N}P_{\;ji}I_{j}(t)+(1-D)I_{i}(t)\\ R_{i}(t+1) & =D\sum_{\;j=1}^{N}P_{\;ji}R_{j}(t)+(1-D)R_{i}(t) \end{array}.$$

### Grid-joint isolation strategy

Since the exclusion of 20,126 SLs during the prelockdown phase constitutes a negligible fraction (0.257%) of the nearly fully connected mobile network, which encompasses 7,819,894 connections, a grid-joint isolation scenario is established under the following conditions to quantitatively evaluate the impact of SLs in identifying potentially infected grids:

1. A grid is preemptively or passively isolated, once any of its neighboring grids is confirmed as infectious and is directly or actively isolated;
2. Individuals destined for isolated grids will remain at home rather than travel to other noninfected grids;
3. Various actively isolated grids may simultaneously opt for passive isolation of their shared neighboring grids, excluding grids that were previously isolated.

Adopting these premises, a grid-joint isolation strategy for NPIs is introduced. Initially, empirical data-derived case triggers the epidemic propagation without intervention until the isolation criteria are met. Grids with infections exceeding *α* are designated as infectious, resulting in the quarantine of all residents and the restriction of routes to and from these grids. Furthermore, unconfirmed grids with possible imported patients are simultaneously subjected to passive isolation, along with travel limitations related to these grids. This isolation process necessitates the recalculation of the mobility matrix among grids and the reassignment of the population across the S, E, I, and R states within each grid. Following this recalibration, the SEIR compartmental epidemic model is applied to update infection dynamics.

### Experimental settings

#### Baseline parameters

In the reaction process of the RDT model, the transmission rate *β* is estimated as the ratio of the effective reproduction number ($R_{t}$) and the mean infectiousness period ($d_{I}$), i.e. $\beta =R_{t}/d_{I}$; the incubation rate *σ* is the inverse of the mean incubation period ($d_{E}$), i.e. $\sigma =1/d_{E}$; the removal rate *r* is expressed as the inverse of the mean infectiousness period, i.e. $r=1/d_{I}$. According to the prior work (57), we set $R_{t}=3.4$, $d_{I}=5.6$, and $d_{E}=1.2$. In the diffusion process, we assume that everyone is willing to start a travel and that they will be infected once they encounter an infectious individual during the prelockdown phase, i.e. $D_{\mathrm{pl}}=1$ and $v_{\mathrm{pl}}=0$. During the lockdown phase, certain movements within the city are still necessary to maintain essential functions and services, leading to $D_{l}=0.2$. The cautious protection measures, such as wearing masks and maintaining good hygiene, lead to increasing immunity, i.e. $v_{l}=0.063$. In the gird-joint isolation strategy, we set the isolation threshold α=3, indicating early intervention.

#### Experimental design

Our numerical analysis investigates the efficacy of SLs in identifying unconfirmed infectious grids and their crucial role in decelerating epidemic spread. To differentiate the effect of SLs from STs, LGs, and SHs in curtailing epidemic propagation, we define a fixed number of ties, *k*, as that of SLs for each grid. Specifically, we extract ties characterized by the top-*k* maximum flow, maximum distance, and minimum distance, respectively. Additionally, we dynamically adjust passively isolated grids based on the infectious PR, which quantifies the likelihood of grid infection (refer to Supplementary Note S2 for details). For comparison, we establish a baseline scenario without grid-joint isolation (NU), relying solely on active isolation.

To minimize the impact of infection dynamics on grids not associated with SLs, we create a new mobility network comprising 4,358 SL-related grids and their 6,172,854 corresponding population flows. Moreover, to assess the relative efficacy of various tie types in reducing infections, we standardize the number of controllable neighbors for each grid to align with the SL scenario. Subsequently, our analysis focuses on selecting passively isolated neighbors for each infected grid. All simulated epidemic propagations are initiated based on actual infection cases before March 5. The epidemic state of each grid when the first active isolation occurs under the SL scenario, serves as the starting point for epidemic propagation simulations for other types of ties, concentrating on the impact of various passive isolations on the differentiation of epidemic propagation.

Through numerical experiments, we explore the deceleration of disease transmission under various isolation strategies. The results are supported by 50 simulations, each simulating disease spread over 90 days. We concentrate on cumulative infections to gauge the impact of ties on mitigating disease spread. The number of restricted grids, routes and populations serves to quantify the cost of quarantine. For every grid-joint isolation process, we document the causal relationships between active and passive isolations among grids to elucidate epidemic control mechanisms. Moreover, to assess the robustness of our findings, we conduct experiments across a broad spectrum of model parameters, i.e. α=5,10,15,20 and $f_{t}=28,56,84,112$. Epidemic propagation from a single initial seed is introduced to explore the effects of the grid feature and patient number on the outperformance of SLs.

## Supplementary Material

pgae515_Supplementary_Data

## Data Availability

Data on the mobility and newly confirmed infections during the preoutbreak, prelockdown, and lockdown phases, as well as the simulation codes are available on Github (https://github.com/JHMou/Strong-long-ties-facilitate-epidemic-containment-on-mobility-networks-mobility-data) for reproducibility of the results in this article.
