# Supplementary material for: Strong long ties facilitate epidemic containment on mobility networks
Source: PNAS Nexus. 2024 Nov 15;3(11):pgae515. doi: 10.1093/pnasnexus/pgae515 (PMC11589786; doi:10.1093/pnasnexus/pgae515)
Supplement: pgae515_Supplementary_Data [file pgae515_supplementary_data.docx]

**
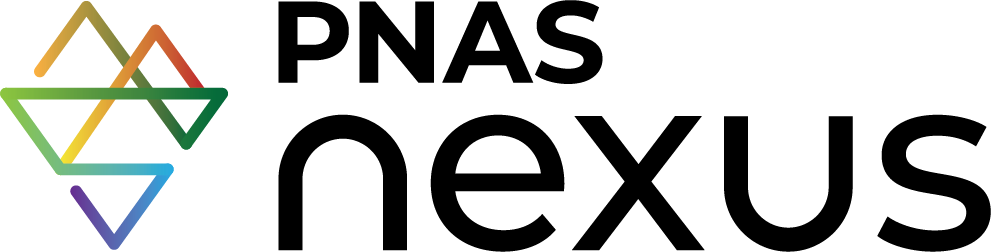
**

**Supplementary Information for**

Strong long ties facilitate epidemic containment on mobility networks

Jianhong Mou^1,+^, Suoyi Tan^1,+^, Juanjuan Zhang^2,3+^, Bin Sai^1^, Mengning Wang^1^, Bitao Dai^1^, Bo-Wen Ming^2^, Shan Liu^4^, Zhen Jin^5^, Guiquan Sun^5,6^, Hongjie Yu^2,3,7 *^, Xin Lu^1, *^

* Corresponding author: Xin Lu and Hongjie Yu

Email: [xin.lu.lab@outlook.com](mailto:xin.lu.lab@outlook.com) and [yhj@fudan.edu.cn](mailto:yhj@fudan.edu.cn)

**This PDF file includes:**

Supplementary text

Figures S1 to S10

Tables S1 to S4

**Supplementary Information Text**

**Supplementary Note 1. Global Moran’s index.** Global Moran’s index is employed to quantify the degree to which similar attributes tend to cluster together spatially. It considers both the spatial proximity of the grids and the similarity in terms of their specific attributes, i.e., the number of incoming connections reached through various types of ties, $z$. The attribute similarity on grid *i* and *j* is equivalent to the covariance between the values of a pair of grids $c_{ij}=(z_{i}-\bar{z})(z_{j}-\bar{z})$, where $\bar{z}$ denotes the mean of the attribute values. The spatial proximity is quantified by the reverse Euclidean distance, i.e., $w_{ij}=1/d_{ij}$. Moran’s index, $\eta$, is then designed to a fixed range as follows:

$$\eta=\frac{\sum_{i=1}^{n} \sum_{j=1}^{n} w_{ij}c_{ij}}{s^{2}\sum_{i=1}^{n} \sum_{j=1}^{n} c_{ij}}$$

Here, $s^{2}$denotes the sample variance:

$$s^{2}=\sum_{i=1}^{n} (z_{i}-\bar{z})^{2}/n$$

If neighboring grids have similar attributes than expected, $\eta>0$. Conversely, if they tend to have more dissimilar attributes, $\eta<0$. When attribute values are spatially distributed randomly and independently, $\eta=0$.

**Supplementary Note 2. Infection pressure.** Infection pressure describes the probability of grid being infected. We calculate the infection pressure $P_{j}(t)$ of grid $j$ according to (3), in which $c_{i}(t)$ is the number of reported cases in grid $i$ on day $t$:

$$P_{j}(t)=\sum_{i,i\neq j}^{n} \left[p_{ij}\sum_{k=1}^{7} c_{i}(t-k)\right]$$

We assumed that the number of infectious individuals in grid $j$ is proportional to the cumulative number of infections during the preceding 7 days. In addition, the movement probability $p_{ij}$ is representative of the movements of infectious individuals.

**Supplementary Note 3. The relationship between community structure and the four types of ties.**

To explore the relationship between four types of ties and community structure in mobility networks, we utilized the Louvain method for community detection. Due to the high mobility between short-distance grids, SHs and STs often connect grids within the same community, with over 50% being intra-community links. Remarkably, 2,780 out of 20,126 SLs are identified as intra-community connections, even though the grids are hundreds of miles apart. LGs, defined as the top-*k* ties with the greatest spatial distance, primarily connect grids at the city periphery. These peripheral grids belong to different communities, making LGs predominantly inter-community connections, with only 2 identified as intra-community links (see Figure 2). SLs capture more inter-community links than SHs and STs, thereby isolating more grids with diverse topological features under the joint-grid isolation strategy. Although LGs connect grids across different communities, most passively isolated grids are located at city peripheries without any patients, thereby reducing the effectiveness of passive isolation.


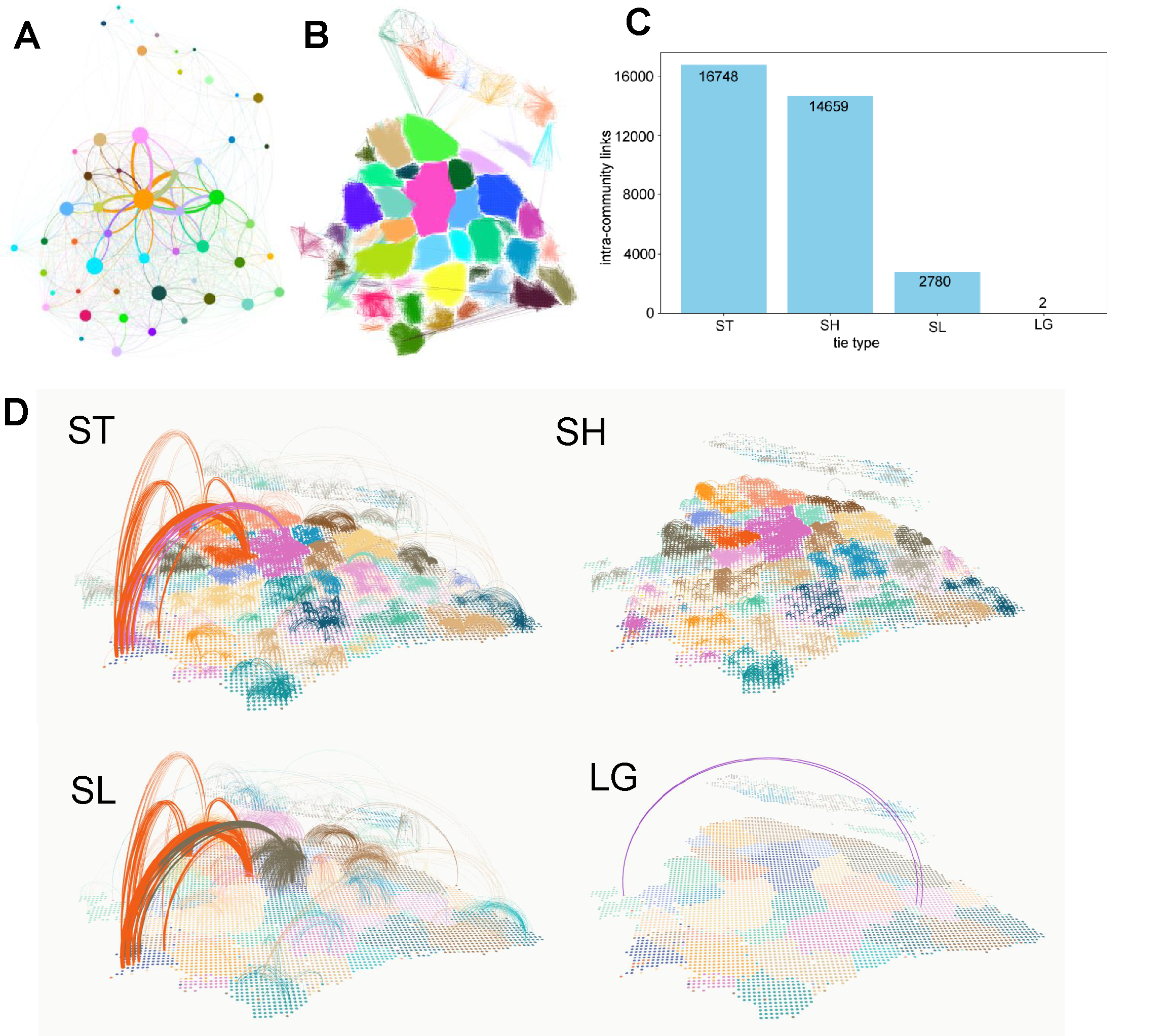


**Figure S1. The relationship between community structure and four types of ties on mobility networks.** **a** displays population flows between communities during the pre-lockdown phase. Each node represents a community, with its center aligned with the centroid of the community. The size of each node is proportional to the area (number of cells) of the community. The width of the directed arrow is proportional to the flows between communities, and the color corresponds to the origin nodes. **b** depicts population flows within the community. The color of the edges indicates flows occurring within the same community, while flows between different communities are filtered. **c** shows the number of intra-community links encapsuled in four types of ties. **d** illustrates the spatial distribution of intra-community links as presented in **c**. Different colors are used to distinguish communities and their respective intra-community links.

**Supplementary Note 4. Identification of strong long ties across China’s mainland.**

To examine the significance of strong long ties in a nation-level mobility network, we extend our experiment to identify strong long ties across China’s mainland. The mobility data utilized in this study was sourced from Cellular Signaling Data (CSD) between 357 cities, spanning from January 1 to January 9, 2020, and aggregated into city-level mobility matrices on a daily basis. We set the corresponding thresholds at the average values, i.e., *d* > 1500 km and *f* > 3576. Our findings indicate that outliers or strong long ties are absent in 98.6% of cities. This conclusion holds even when considering a narrower range of distance and flow, such as *d* > 1000 km and *f* > 500 (see Figure 1).

The different internal drivers of trips for individuals within cities and between cities account for this discrepancy. Specifically, grids within cities are more heterogeneous due to the scattered distribution of points of interest (POIs), which are easily accessible within short commutes at a lower cost. Conversely, the adequate infrastructure within each city, i.e., the homogeneity in basic living standards, and the high cost of long-distance travel reduce the necessity for most people to travel between cities over several hundred miles. The varying significance of SL between city-scale and national-level mobility networks serves as a reminder of Simpson's Paradox and underscores the importance of our work.


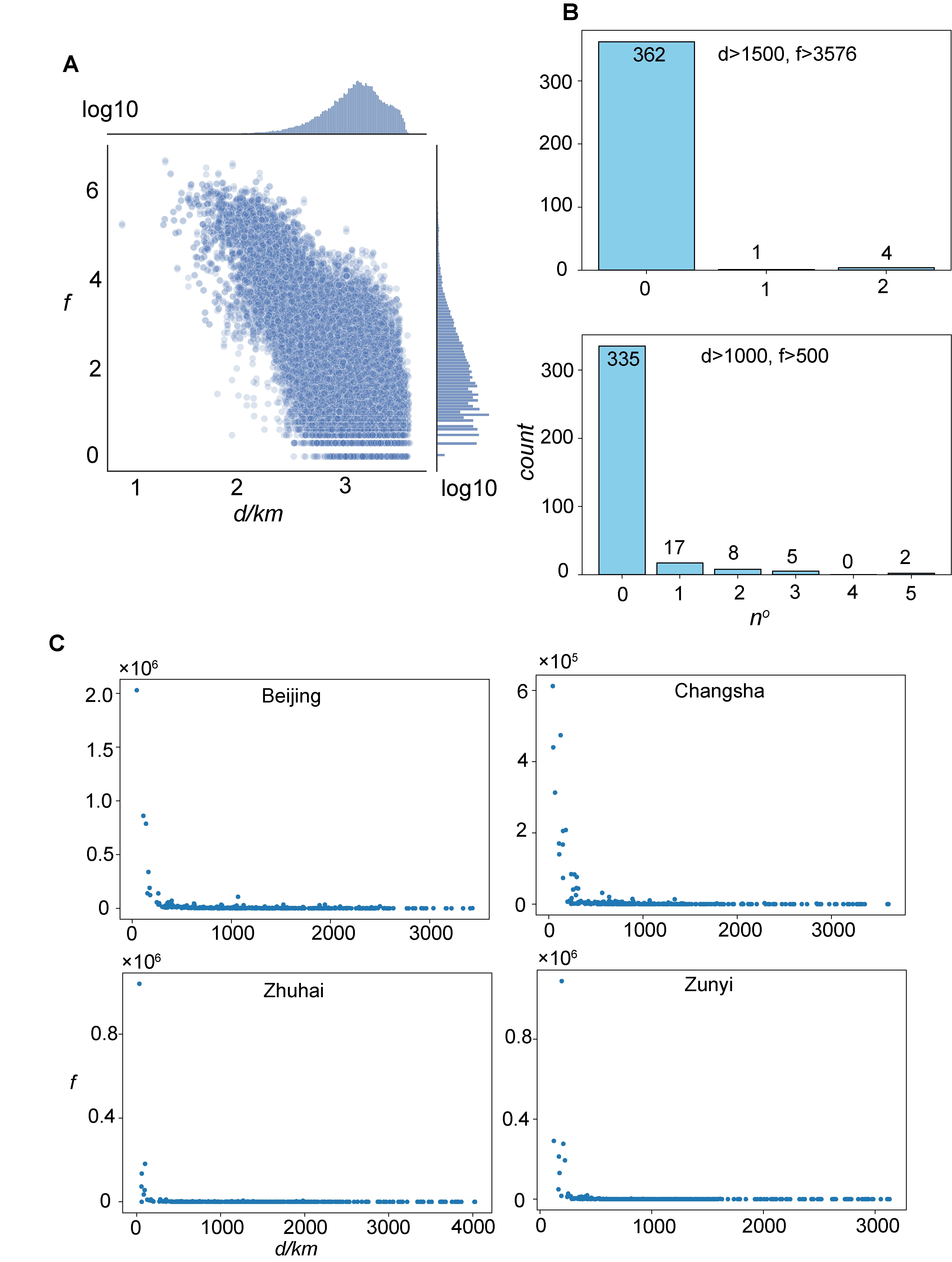


**Figure S2. The joint distribution of distance and flow for cities within the national-scale mobility network. a** depicts the joint distribution using a double log10 axis. **b** presents a histogram showing the number of cities with varying numbers of strong long ties, i.e., $n^{o}$. **c** highlights Beijing, Changsha, Zhuhai, and Zunyi, representing cities at various stages of development.

**Supplementary Note 5. DBSCAN for identifying strong long ties.** For quantitative identification, we define strong long ties (SLs) as connections with a flow surpassing that of spatially proximate ties from the same origin, essentially identified as the outliers in the joint distribution of distance and flow for each grid. By setting the radius of the neighborhood ($\varepsilon=0.02$) and the minimum number of points required to form a dense region ($s=5$), DBSCAN begins with an arbitrary point and retrieves its $\varepsilon$-neighborhood. If there are at least $s$ points within this neighborhood (designated as core points), a new cluster is initiated. The algorithm then recursively explores all points in the $\varepsilon$-neighborhood, expanding the cluster by including all reachable core points and their neighbors. If a point is not a core point, the algorithm proceeds to the next unvisited point. This process continues until all points have been visited. Ultimately, all points are classified as either part of a cluster or as noise.


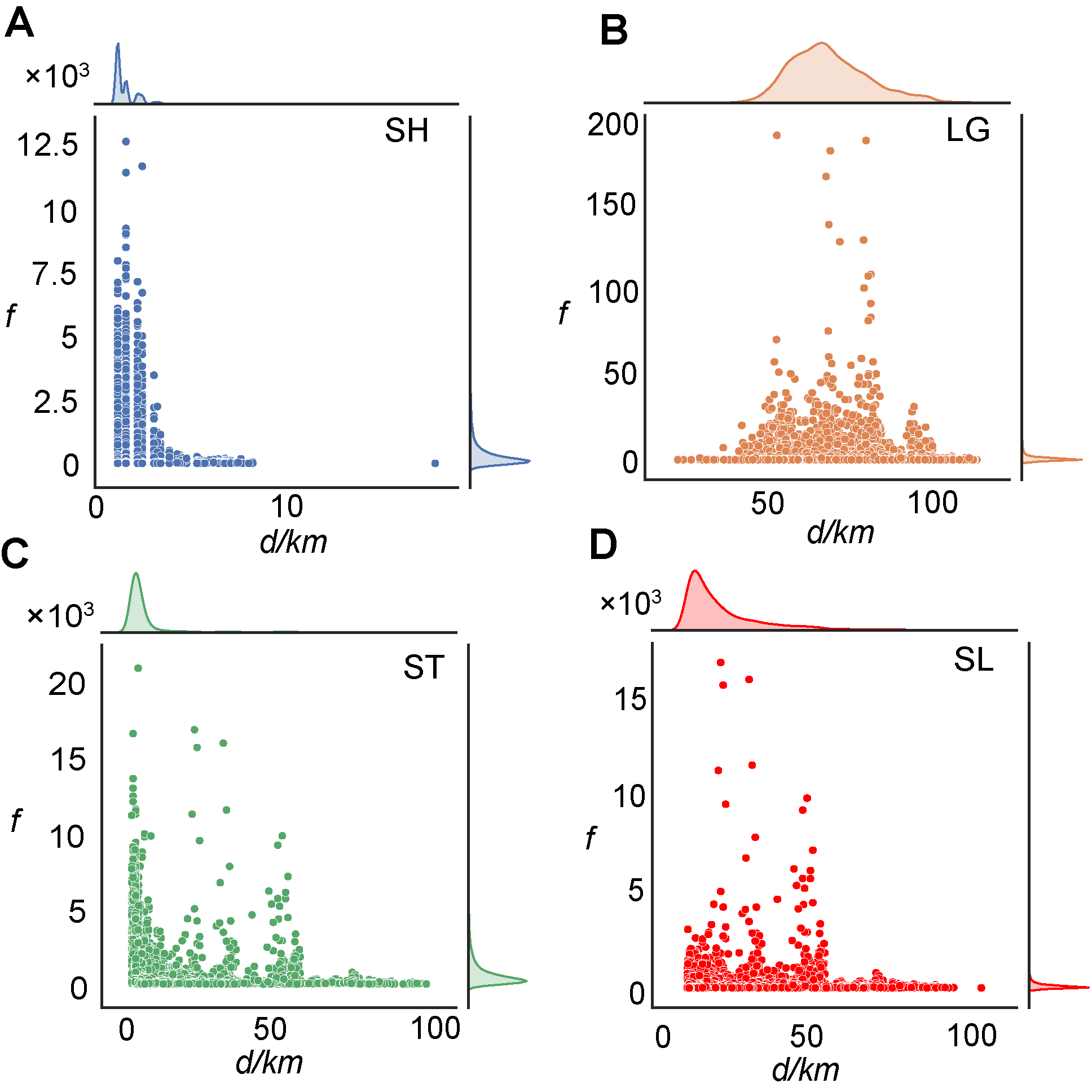


**Figure S3. The joint distribution of flow and distance of ties during the pre-lockdown phase.** STs predominantly concentrate on grid pairs with average distance $\bar{d}=5656.93$ and average flow $\bar{f}=810.65$. SLs concentrate on outliers with a relatively narrow flow distribution. LGs, covering distances from 50 to 100km, typically demonstrate flows under 200. SHs, excluding self-connections, focus on local connections within a 10km radius, with an average flow $\bar{f}=404.92$.


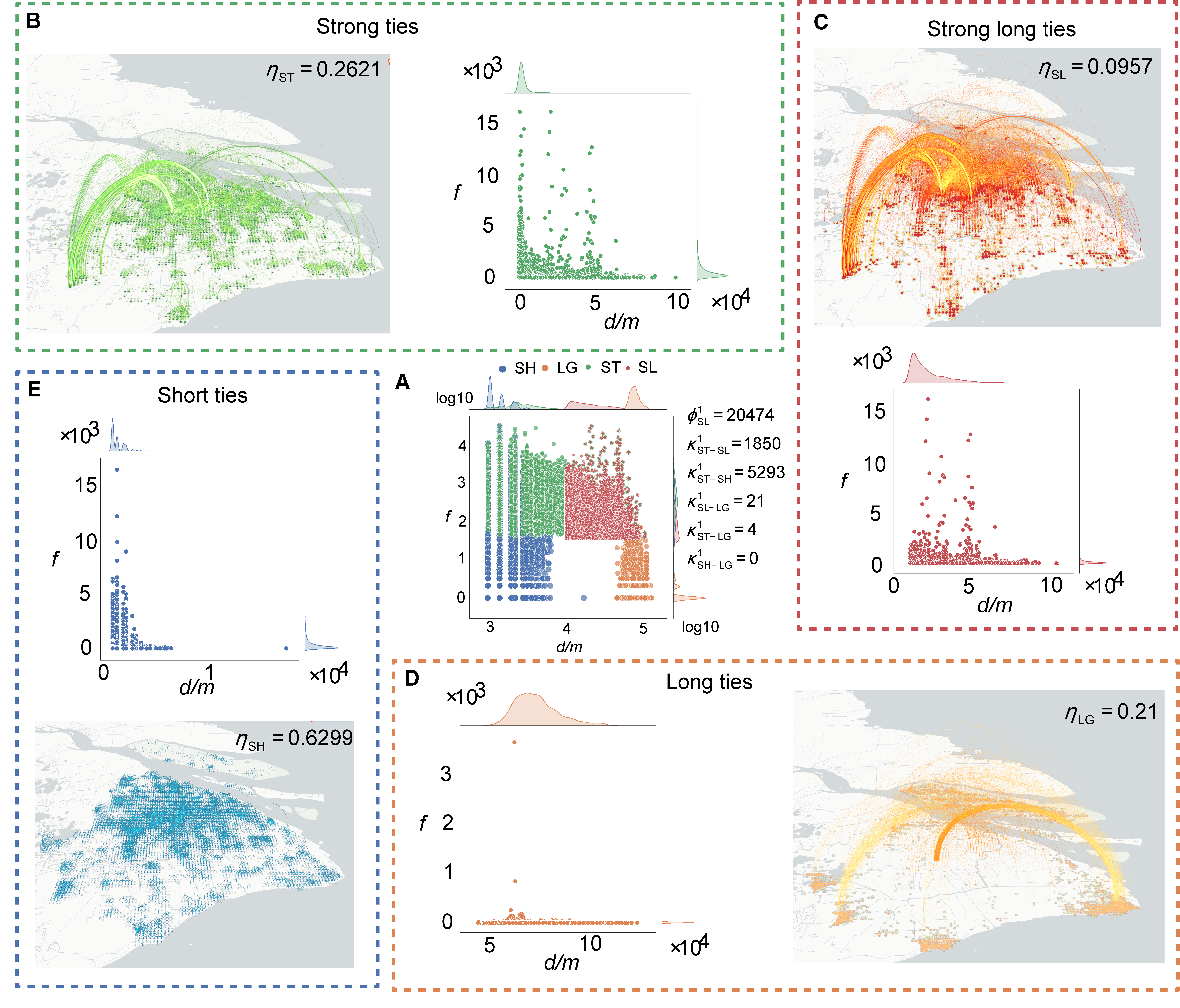


**Figure S4. Statistical and spatial characteristics of various types of ties during the pre-outbreak phase.** Although different types of ties share several ties, they show various statistical and spatial characteristics. (a) illustrates the relationship between distance and flow of STs, LGs, SLs and SHs. The number of all types of ties for each grid keeps in line with that of SLs. is the number of SLs during the pre-outbreak phase and $\kappa_{ST-SL}^{1}$ represents the number of overlapping ties between STs and SLs. 25.85% and 9.04% of STs are also SHs and SLs respectively, while only 0.1% of SLs are simultaneously identified as LGs. (b-e) respectively exhibit the joint distribution of distance and flow, and the spatial distribution of STs, SLs, LGs, and SHs along with Moran’s indices. Linewidth represents the strength of the connection.


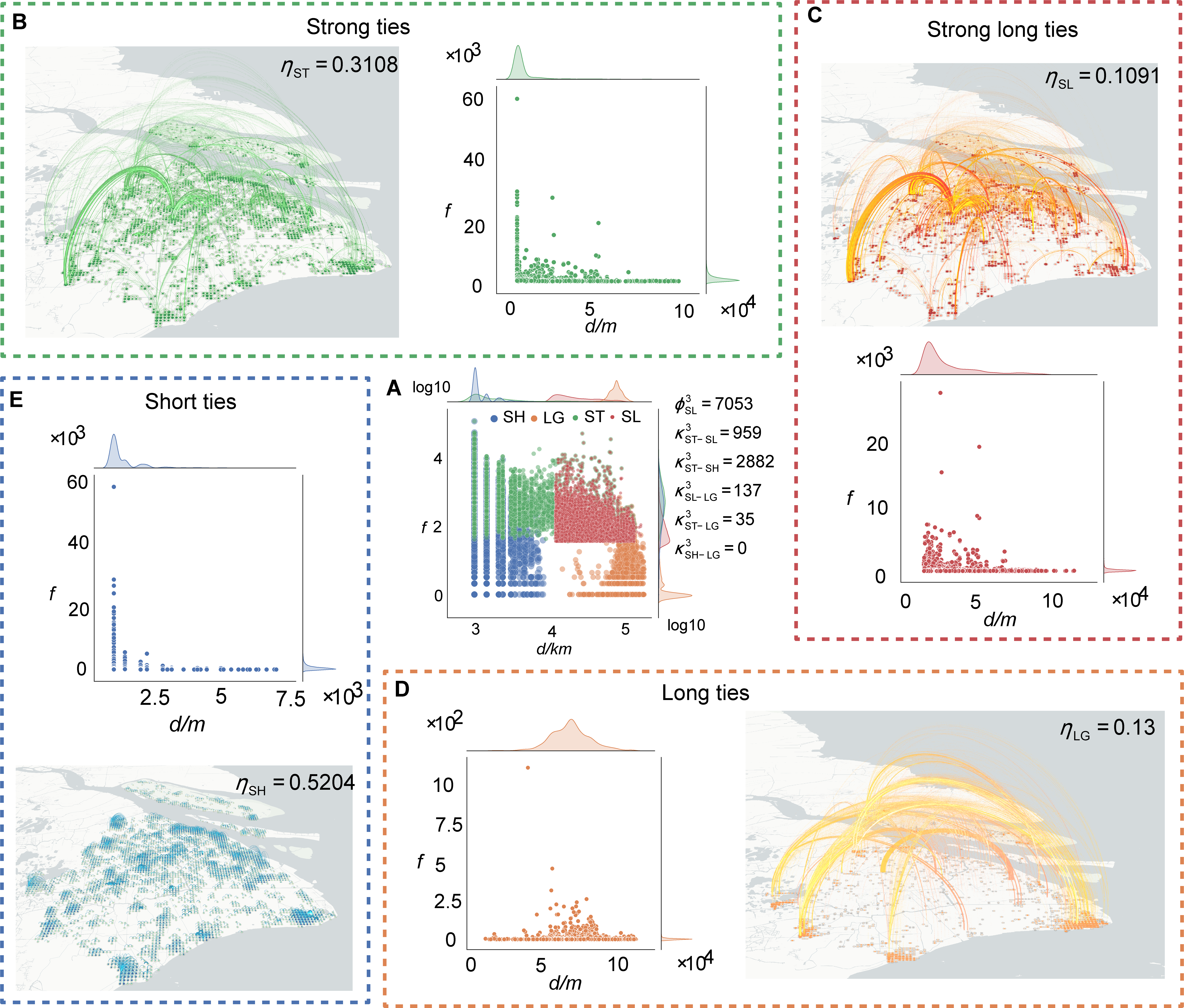


**Figure S5. Statistical and spatial characteristics of various types of ties during the lockdown phase.** (a) illustrates the relationship between distance and flow of STs, LGs, SLs and SHs during the lockdown phase. 40.86% and 13.6% of STs are also SHs and SLs respectively, while only 1.94% of SLs are simultaneously identified as LGs. (b-e) respectively exhibit the joint distribution of distance and flow, and the spatial distribution of STs, SLs, LGs, and SHs along with Moran’s indices.


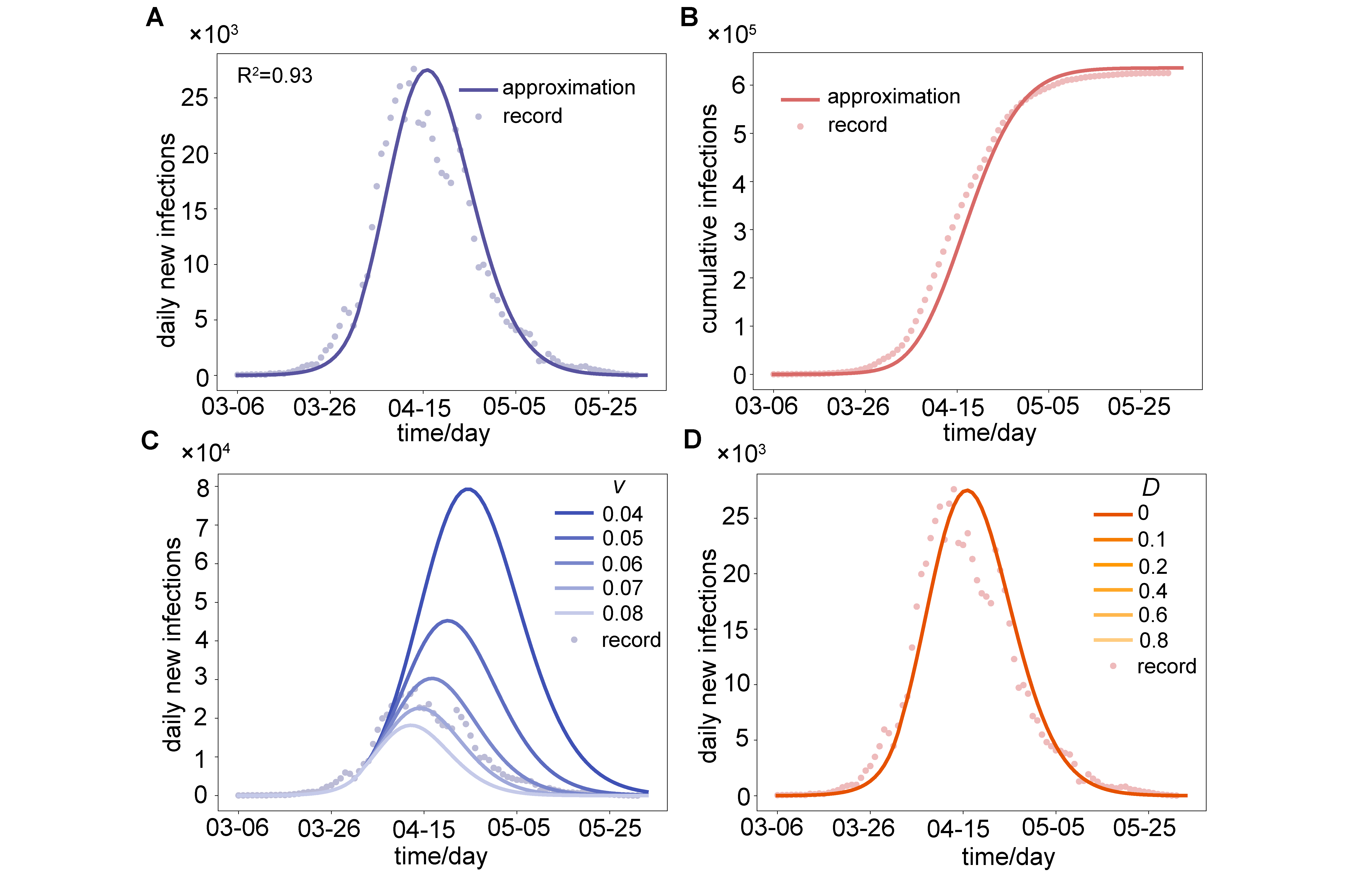


**Figure S6. The performance of the reaction-diffusion model on approximating epidemic propagation in the mobility network.** RDT model is capable of reproducing the daily new infections with the goodness of fit $R^{2}=0.93$. (a) and cumulative infections (b) of COVID-19 in Shanghai. It is sensitive to the immunity probability $v$ (c) and robust to the exit probability $D$ (d).


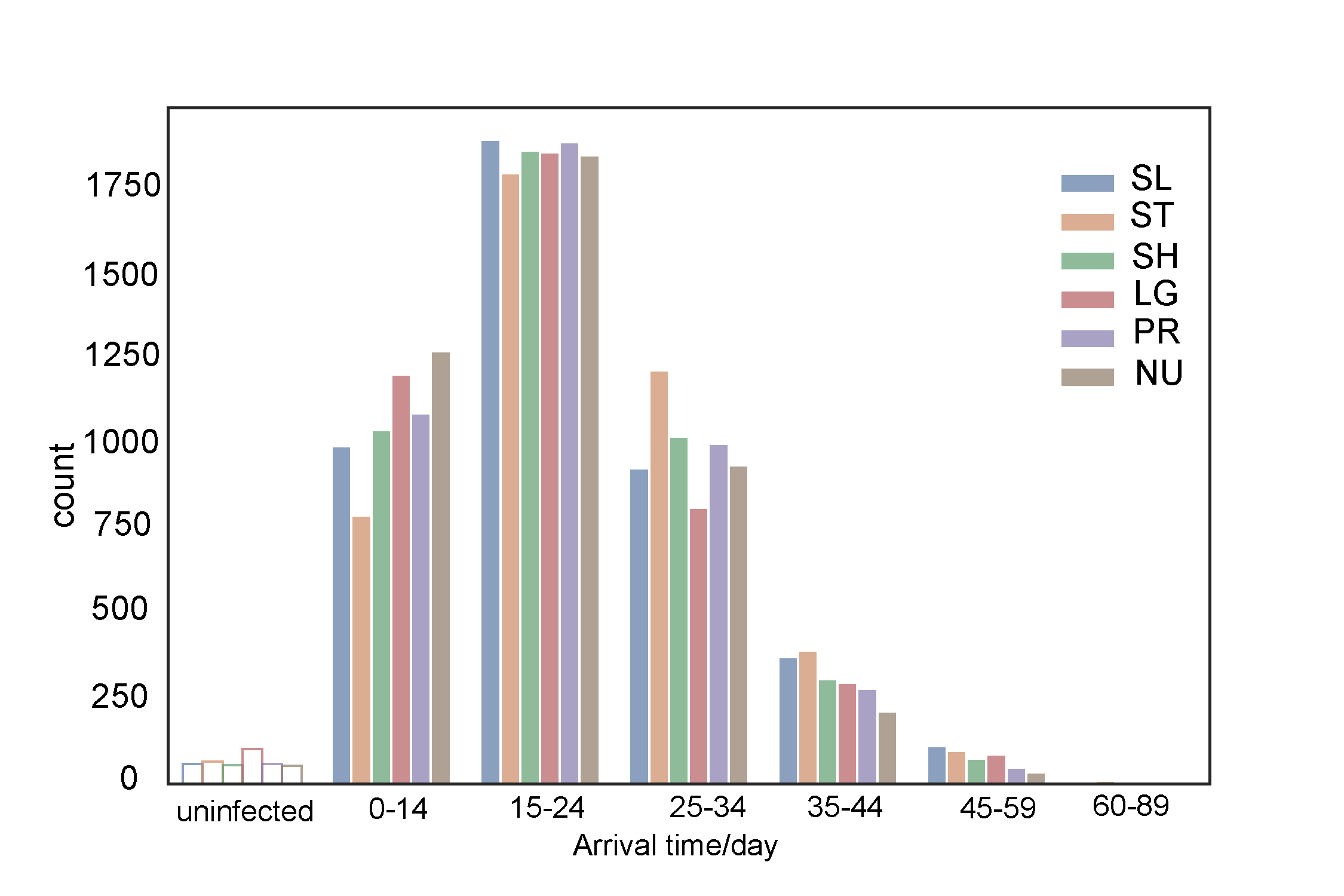


**Figure S7. The statistical distribution of arrival time for different control measures.** Arrival time is separated into 7 classes where the number of uninfected grids is represented as hollow histograms. Noticeably, LGs witness the most uninfected grids since the passive isolated grids are usually located on the city periphery without any patients when they are isolated in advance.





Figure S8. The dynamics of daily confirmed infections for different control measures. Although SL-oriented grid-joint isolation yields more infections than that of ST at *t*=35, it keeps a low level since *t*=50. In the context of STs, several grids with arrival time smaller than 14 days are identified as satellite seeds and initiate new outbreaks, raising the number of grids with an arrival time of 25-34 days. The reaction process of the RDT model within these grids induces increasing infections after several days. The color bar quantifies the daily confirmed infections of grids.


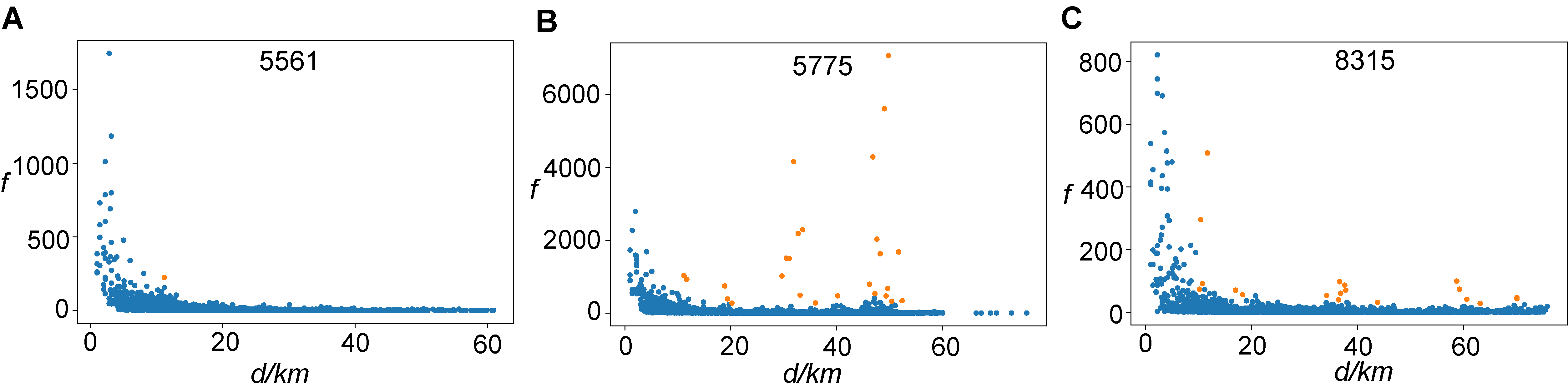


Figure S9. Grids with distinct features of SLs. Grid 5561 illustrates few non-significant SLs; grid 5775 displays several SLs with larger flow than SHs; grid 8315 shows significant SLs displaying smaller flow than SHs.


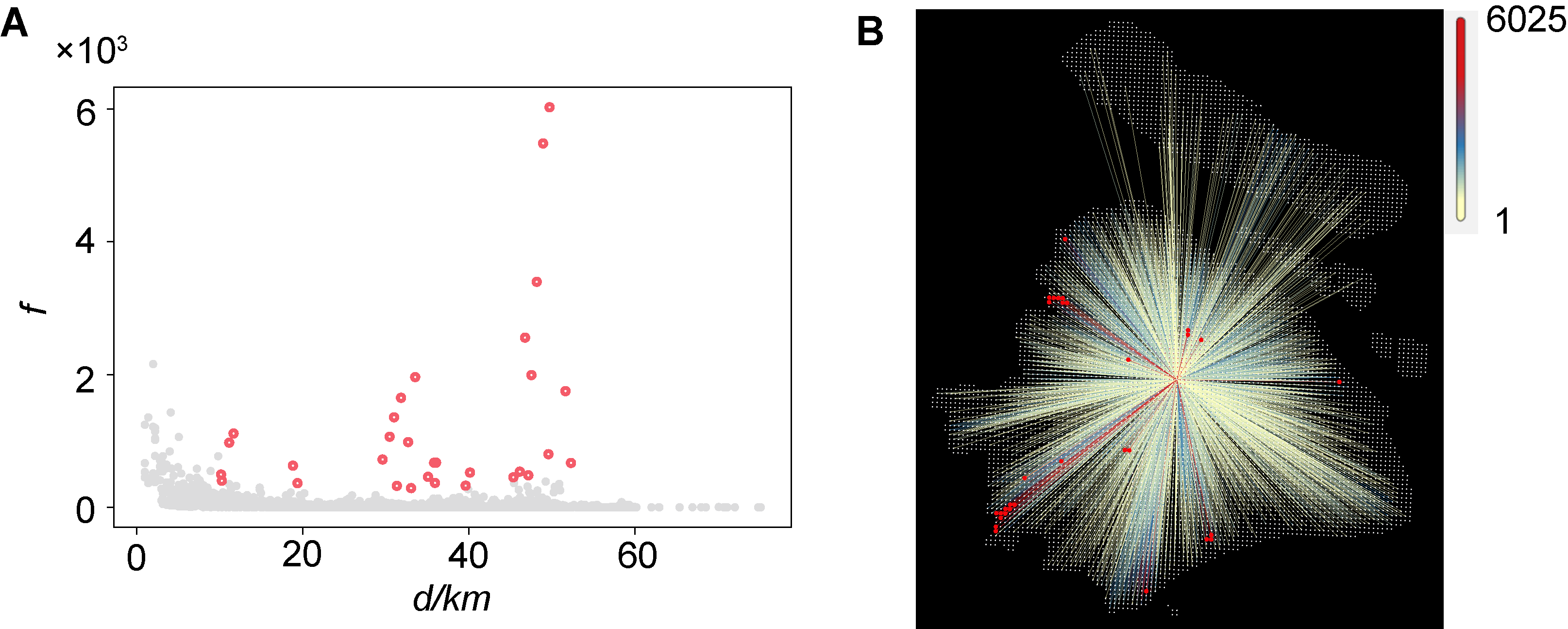


**Figure S10**. **Strong long ties of Shanghai South Railway Station during the pre-outbreak phase.** Several SLs yield heavier population flow than SHs. (a) shows the joint distribution of ties departing from Shanghai South Railway Station, where the red circle denotes the detected SLs. (b) represents the spatial distribution of these ties, where red points are the terminals of SLs. Shanghai South Railway Station serves a larger number of passengers from distant locations compared to the nearby vicinity.

**Table S1. Summary of the basic topological features of the mobility networks across phases.**

| **Network** | ***N*** | ***M*** | ***<w>*** | ***C*** | ***r*** | ***<k^in^>*** | ***T*** | ***E*** | ***A_s_*** |
| --- | --- | --- | --- | --- | --- | --- | --- | --- | --- |
| Pre-outbreak | 5506 | 555893 | 130.57 | 0.60 | 0.16 | 100.96 | 0.47 | 0.34 | 3.75 |
| Pre-lockdown | 6123 | 751192 | 154.65 | 0.56 | 0.26 | 122.68 | 0.47 | 0.34 | 1.78 |
| Lockdown | 6426 | 263716 | 215.23 | 0.57 | 0.17 | 41.04 | 0.43 | 0.26 | 1.42 |

Note: Nodes (*N*) represent the grids visited, and edges (*M*) represent trips between grids. <*w*> is the average weight. *C* and *r* are the clustering coefficient and the assortative coefficient, respectively. <*k^in^*> is the average in-degree. *T* and *E* are the transitivity and global efficiency, respectively. *A_s_* denotes the directional asymmetry that is quantified by the variation of in-degree and out-degree, i.e., $A_{s}=\sum_{i=1}^{N} (k_{i}^{out}-k_{i}^{in})^{2}$.

**Table S2. Spatial autocorrelation analysis of different types of ties during various phases.**

| **Phases** | **Tie type** | $\eta$ | ***Z-score*** |
| --- | --- | --- | --- |
| Pre-outbreak | SLs | 0.0957^*^ | 18.411 |
|  | SHs | 0.6299^*^ | 104.628 |
|  | STs | 0.2621^*^ | 43.924 |
|  | LGs | 0.21^*^ | 35.778 |
| Pre-lockdown | SLs | 0.0873^*^ | 15.894 |
|  | SHs | 0.5342^*^ | 88.738 |
|  | STs | 0.1879^*^ | 31.302 |
|  | LGs | 0.2406^*^ | 40.876 |
| Lockdown | SLs | 0.1091^*^ | 19.2 |
|  | SHs | 0.5204^*^ | 86.471 |
|  | STs | 0.3108^*^ | 51.8 |
|  | LGs | 0.13^*^ | 23.11 |

Note: *** represents the significance of $\eta$, i.e., *p*-value<0.001; *z*-score denotes the multiply of standard deviation. There is a clustered distribution if *z*>1.65.

**Table S3. Number of grid pairs with the gap of arrival time smaller than 7 days.**

| **Phase of ties** | **Phase of epidemics** | $n_{sl}$ | $n_{sh}$ | $n_{st}$ | $n_{\lg}$ | $r_{sh}$ | $\left\langle r_{sh}\right\rangle$ | $r_{st}$ | $\left\langle r_{st}\right\rangle$ |
| --- | --- | --- | --- | --- | --- | --- | --- | --- | --- |
| Pre-outbreak | Pre-lockdown | 1636 | 2866 | 2781 | 85 | 0.5708 | 0.6023 | 0.5883 | 0.6139 |
| Pre-lockdown | Lockdown | 3508 | 5536 | 5485 | 44 | 0.6337 |  | 0.6396 |  |
| Pre-lockdown | Pre-lockdown | 1372 | 2137 | 2146 | 184 | 0.6420 | 0.7126 | 0.6393 | 0.7177 |
| Lockdown | Lockdown | 1362 | 1739 | 1711 | 230 | 0.7832 |  | 0.7960 |  |

Note: $n_{sl}$, $n_{sh}$, $n_{st}$ and $n_{\lg}$ respectively represent the number of grid pairs with an arrival time gap smaller than 7 days connected by SLs, SHs, STs and LGs. $r_{sh}$and $r_{st}$ respectively denote the ratio between $n_{sl}$ and $n_{sh}$, $n_{st}$, i.e., $r_{sh}=n_{sl}/n_{sh}$. means the average value.

**Table S4. The cumulative infections under various containment strategies.**

| **Strategy** | $I_{c}$ | $R_{d}$ |
| --- | --- | --- |
| Grid-joint isolation based on SLs | 430680.94 | / |
| Grid-joint isolation based on SHs | 514766.33 | -0.163 |
| Grid-joint isolation based on STs | 515156.27 | -0.164 |
| Grid-joint isolation based on LGs | 514313.70 | -0.162 |
| Grid-joint isolation based on infection pressure | 519806.10 | -0.171 |
| No interventions | 535806.30 | -0.196 |

Note: $R_{d}$ denotes the ratio between the cumulative infections of SLs and others, i.e., $R_{d}^{ST}=(I_{c}^{SL}-I_{c}^{ST})/I_{c}^{ST}$.
